# Scaling Nurturing Care Interventions in the Health Sector: A Theory of Change Perspective

**DOI:** 10.3389/fpubh.2022.903342

**Published:** 2022-06-13

**Authors:** Muneera A. Rasheed

**Affiliations:** Department of Paediatrics and Child Health, The Aga Khan University, Karachi, Pakistan

**Keywords:** nurturing care, theory of change (ToC), private sector, integration, scale-up

## Abstract

Nurturing care interventions postulated on strengthening caregiver-child relationships have proven to be effective for improving early childhood development outcomes in low- and middle-income countries. Hence, a scale-up of the interventions has been recommended with an emphasis on the health sector given the contact with families in the critical first 3 years of life. However, an effective scale-up of an integrated intervention through healthcare requires a theory of change approach elucidating pathways of sustainable change. From this viewpoint, I reflect on my experience of scaling the intervention in a private pediatric care setting. I realized that buy-in from the health sector required realization of benefits to include health outcomes framed as the potential to improve the quality of life and the process of recovery; sustainable behavior change required a culture that promoted nurturing care highlighting the role of leadership; subsequently improving the experience of frontline staff and at an individual level, this could be achieved through the provision of supportive supervision-rooted in a framework of compassion. The lessons learned are shared to be considered for future integration efforts.

Nurturing care (NC) is defined as a “stable environment created by parents and other caregivers that ensures children's good health and nutrition, protects them from threats, and gives young children opportunities for early learning, through interactions that are emotionally supportive and responsive” (1). It has been recommended as a crucial strategy to mitigate the effect of over 250 million children (43%) younger than 5 years in low- and middle-income countries (LMICs) estimated to be at increased risk of not achieving their maximum human potential, with long-lasting impact on adult productivity (2). Health programs can be leveraged as an entry point to scale NC intervention given the extensive reach to these families during the critical early years. Moreover, there is documented evidence of feasibility and effectiveness of integrating additional components of responsive caregiving and stimulation at the point of delivery. However, case studies detailing the implementation process of institutionalizing the integrated intervention remain scarce. In this perspective, I start with a brief description of the assumptions which need to be realized for the desired outcomes of integration to be achieved. Then taking an empathetic approach, I list the challenges of the health sector from their perspective and suggest a way forward that takes their genuine concerns into account (being sensitive). The core strategy was adopting the principles of NC to create an enabling system that allows the frontline staff to experience being nurtured (being responsive). I build this narrative from my experiences of integrating the NC intervention [Care for Child Development (3) module] in a private tertiary-care hospital in Pakistan.

## Theory of Change

**Figure 1:**
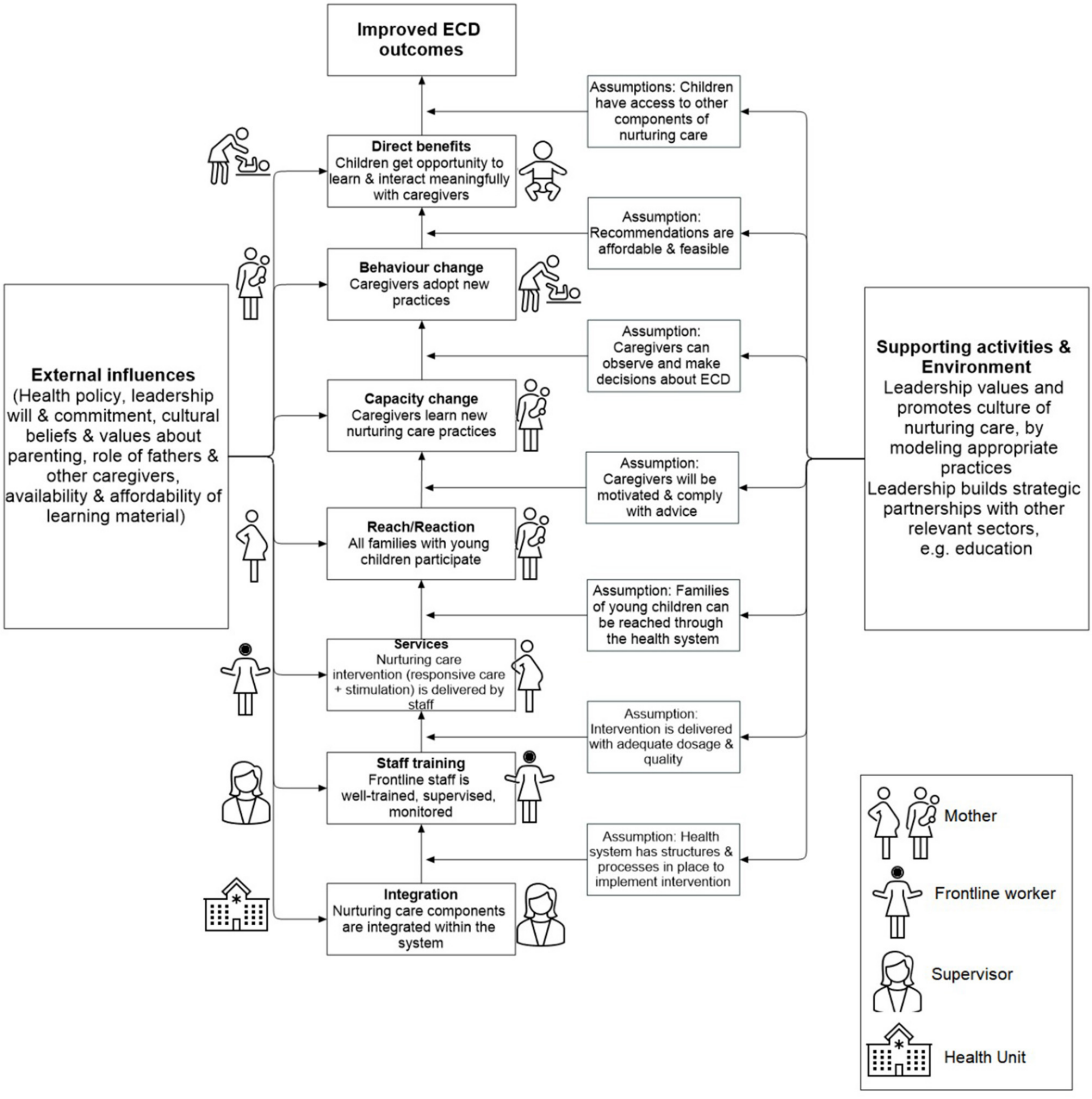
A recommended theory of change for integrating nurturing care intervention in health systems.

A ToC (4) model of integrating NC in a health system for the goal of improved early childhood development (ECD) outcomes has four broad areas of focus for behavior change: the mother, the frontline worker, her supervisor, and the health system (Figure 1). The assumptions for the changes in mothers' behaviors are that they are motivated to improve their children's development once they receive new knowledge and learn new ways to strengthen their parenting skills and, importantly, have the autonomy to do so. The motivation for the frontline worker to change her behavior assumes that the health system can engage the worker for delivery of the intervention through a robust supervision system. The supervisory support would ensure the required dosage was delivered (professional accountability), with adequate quality (enhancing competence), while also treating her as an individual with respect (emotional experience with the work). While there is sufficient evidence around interventions to realize the assumptions related to supervisory support (5), further studies are required to operationalize the interventions for the supervisor and the overall system (at-risk assumptions). The at-risk assumption related to the supervision system is that support strategies for the supervisor/manager are in place along the same principles. These strategies need not just training for the new skill set but are also modeled and encouraged by the higher leadership. The final at-risk assumption with respect to the health system is that supervision pertaining to NC sustains because a culture that values NC is promoted.

## The Challenges

There were multiple challenges when scaling up an NC intervention model which is a behavior change intervention through the health sector. Securing buy-in of healthcare leaders when framing the argument as a case of long-term benefits for adult productivity was not met with enthusiasm and rightly so. Educational attainment does not fall into the realm of the health sector and adult productivity is a long-term outcome. The health sector is appraised against health outcomes just like the education sector is responsible for academic ones. Hence, when the disease burden is high and resources are limited, the natural course of action is to prioritize interventions that will improve health indicators.

Secondly, the routine training of health workers (especially nursing) involved giving didactic messages emerging from a traditional medical model though compassionate care is highly encouraged in medicine. Reframing training of nursing staff to deliver a relationship-based intervention embedded in human empathy required a significant shift of responsibility toward families, to empower them to become partners in the healthcare journey of their child.

A third challenge was that no provision of continued professional education of managers as supervisors existed in both systems–a gap expressed by the participants themselves to ensure the quality of the intervention delivery. An effective supervision characterized by intellectual competence as well as the empathetic approach in relationships is pivotal to an engaged workforce. The skills needed to be built through supervision entailed modeling similar principles of shared decision-making, encouraging dialogue, and mutual respect, creating an experience to flow because of a virtuous cycle.

Leadership practices in healthcare settings in LMIC are generally hierarchical with managers exercising power in a narrow space of decision-making (6). A greater challenge was there was no formal (or informal) training for leadership around skills to engage employees and the positions are usually held based on clinical experience. Such practices affect staff motivation and disengage them. Delivering an intervention that was essentially postulated in human vulnerability would not have been possible for a worker who had never been on the receiving end herself. To ensure quality, the supervision system had to be strengthened and a culture shaped should be valued for promoting relationships and connections, which allowed being sensitive and responsive to nurture the subordinates especially the frontline staff.

## The Strategy

At the macro-level, NC was framed as part of the vision of the health system for provision of quality care (7). Literature from high-income countries has indicated that such interventions can reduce morbidities and improve the process of recovery and the quality of life in children (8). Additionally, the intervention can shift the focus to the development and behavior of every child in the system and a greater multidisciplinary approach to healing. These are important indicators of the quality of healthcare systems (9). Another strategy for framing was to reduce anxieties related to the new task. NC considerably overlaps with the notion of “compassionate” and “empathetic” care which is widely used in healthcare services. When NC was presented along those lines, it was perceived as a familiar concept thereby making people feel like doing something which is supposed to be part of their job.

Framing was followed by a shift toward human-centered practices modeled by those in leadership positions using an innovative social media strategy (10) as an opportunity to mentor the next layer of managers (11). Staff were provided space to express their challenges. Just like with the intervention activities (play) serving as an entry point to enhance the quality of mother/child interaction promoting early attachment and bonding, specific activities were envisioned to promote connections between supervisors and the staff.

At the micro-level, building the capacity of supervisors of the frontline workers through mentorship to ensure optimal delivery of health messages, including NC, formed promising inception. A mentorship program was developed embedded in a framework of compassion, enabling supervisors to connect with the frontline staff for a stronger relationship (12). While this meant extra efforts for training for NC, it also served as an opportunity to bring a new approach to counseling that could positively impact all areas of healthcare counseling. The approach of working with the supervisor and allowing her to mentor the frontline worker was found to gain quick buy-in for efficient implementation.

An important non-monetary incentive for health workers is their relationship with the patients and families they serve. Through this training and a focus on counseling skills, this served as an added incentive for uptake of NC intervention. Strengthened relationships can also be a source of strength for families especially for women in settings who may otherwise have no social support at home.

## Lessons Learned

The experience with the implementation of the projects has yielded lessons that can be of value to others who wish to support the integration of NC intervention.

- The quality of implementation of NC intervention is inherently dependent on the health system from which it operates. Hence, any strategy for scaling up needs to contain an element of system strengthening. Health system experts who can provide insights about health structures and organizational psychologists who understand the processes of human interaction at workplaces as part of the core team should be included.
- Approach the support as a partnership model with internal stakeholders. Recognize their strengths and provide them with an opportunity to co-design and co-lead where possible, e.g., the nursing mentorship package was led by the nursing team. This leads to greater engagement and ownership of the stakeholders important for sustainability.
- Instead of the traditional approach which creates parallel structures through research staff, the focus should be on strengthening and engaging the existing structure of training, supervision and monitoring, and evaluation.
- A theory of change model should be created to design the external support mechanism explicitly laying out the causal pathways to effective and sustainable integration. The approach requires a realistic evaluation of the capacity of the health system and their staff to implement nurture care and then design accordingly since it is not just a new skill but also a new behavior.

## Data Availability Statement

The original contributions presented in the study are included in the article/supplementary material, further inquiries can be directed to the corresponding author.

## Author Contributions

MR review the literature and drafted the perspective.

## Conflict of Interest

The author declares that the research was conducted in the absence of any commercial or financial relationships that could be construed as a potential conflict of interest.

## Publisher's Note

All claims expressed in this article are solely those of the authors and do not necessarily represent those of their affiliated organizations, or those of the publisher, the editors and the reviewers. Any product that may be evaluated in this article, or claim that may be made by its manufacturer, is not guaranteed or endorsed by the publisher.

## References

[1] World Health Organization United Nations Children's Fund World Bank Group. Nurturing Care for Early Childhood Development: A Framework for Helping Children Survive and Thrive to Transform Health and Human Potential. Geneva: World Health Organization (2018). Accessed online at: https://nurturing-care.org/

[2] Lu C Black MM Richter LM. Risk of poor development in young children in low-income and middle-income countries: an estimation and analysis at the global, regional, and country level. Lancet Glob Health. (2016) 4:e916–22. 10.1016/S2214-109X(16)30266-2 27717632 PMC5881401

[3] Lucas JE Richter LM Daelmans B. Care for Child Development: an intervention in support of responsive caregiving and early child development. Child Care Health Dev. (2018) 44:41–9. 10.1111/cch.12544 29235167

[4] Breuer E Lee L De Silva M Lund C. Using theory of change to design and evaluate public health interventions: a systematic review. Implementation Sci. (2015) 11:1–17. 10.1186/s13012-016-0422-6 27153985 PMC4859947

[5] Yousafzai AK Rasheed MA Siyal S. Integration of parenting and nutrition interventions in a community health program in Pakistan: an implementation evaluation. Ann NY Acad Sci. (2018) 1419:160–78. 10.1111/nyas.13649 29791730

[6] Gilson L Agyepong IA. Strengthening health system leadership for better governance: what does it take? Health Policy Plann. (2018) 33:ii1–4. 10.1093/heapol/czy052 30053034 PMC6037056

[7] Rasheed MA Bharuchi V Mughis W Hussain A. Development and feasibility testing of a play-based psychosocial intervention for reduced patient stress in a pediatric care setting: experiences from Pakistan. Pilot Feasibil Stud. (2021) 7:1–13. 10.1186/s40814-021-00781-8 33673877 PMC7936486

[8] Boles J Fraser C Bennett K Jones M Dunbar J Woodburn A. The Value of Certified Child Life Specialists: Direct Downstream Optimization of Pediatric Patient Family Outcomes. Association of Child Life Professionals (2020). Available online at: https://www.denverhealth.org/-/media/files/departments-services/pediatrics/value-of-cclss-full-report.pdf

[9] Kruk ME Gage AD Arsenault C Jordan K Leslie HH Roder-DeWan S. High-quality health systems in the Sustainable Development Goals era: time for a revolution. Lancet Glob. Health. (2018) 6:e1196–252. 10.1016/S2214-109X(18)30386-3 30196093 PMC7734391

[10] Rasheed MA Hookmani AA Waleed S Fatima HS Siddiqui S Khurram M. Implementation and evaluation of a social media-Based communication strategy to enhance employee engagement: experiences from a Children's Hospital, Pakistan. Front Publ Health. (2021) 9:584179. 10.3389/fpubh.2021.584179 33777875 PMC7991406

[11] Rasheed MA Kedzierski JT Hasan BS. Improved family experience outcomes in a pediatric hospital in Pakistan: mentoring, human-centered practice, and theory of change. NEJM Catal Innov Care Deliv. (2021). 2. 10.1056/CAT.21.0099

[12] Hookmani AA Lalani N Sultan N Zubairi A Hussain A Hasan BS. Development of an on-job mentorship programme to improve nursing experience for enhanced patient experience of compassionate care. BMC Nurs. (2021) 20:175. 10.1186/s12912-021-00682-4 34537031 PMC8449216

